# Instrument Variables for Reducing Noise in Parallel MRI Reconstruction

**DOI:** 10.1155/2017/9016826

**Published:** 2017-01-19

**Authors:** Yuchou Chang, Haifeng Wang, Yuanjie Zheng, Hong Lin

**Affiliations:** ^1^Computer Science and Engineering Technology Department, University of Houston-Downtown, Houston, TX 77002, USA; ^2^Massachusetts General Hospital, Charlestown, MA 02129, USA; ^3^Harvard Medical School, Boston, MA 02115, USA; ^4^School of Information Science and Engineering, Institute of Life Sciences, Key Laboratory of Intelligent Information Processing, Shandong Normal University, Jinan 250014, China

## Abstract

Generalized autocalibrating partially parallel acquisition (GRAPPA) has been a widely used parallel MRI technique. However, noise deteriorates the reconstructed image when reduction factor increases or even at low reduction factor for some noisy datasets. Noise, initially generated from scanner, propagates noise-related errors during fitting and interpolation procedures of GRAPPA to distort the final reconstructed image quality. The basic idea we proposed to improve GRAPPA is to remove noise from a system identification perspective. In this paper, we first analyze the GRAPPA noise problem from a noisy input-output system perspective; then, a new framework based on errors-in-variables (EIV) model is developed for analyzing noise generation mechanism in GRAPPA and designing a concrete method—instrument variables (IV) GRAPPA to remove noise. The proposed EIV framework provides possibilities that noiseless GRAPPA reconstruction could be achieved by existing methods that solve EIV problem other than IV method. Experimental results show that the proposed reconstruction algorithm can better remove the noise compared to the conventional GRAPPA, as validated with both of phantom and in vivo brain data.

## 1. Introduction

Over the past few years, generalized autocalibrating partially parallel acquisition (GRAPPA) [1], as an efficient parallel magnetic resonance imaging (pMRI) technique, has been widely studied. However, two main categories of errors exist within GRAPPA method: model error and noise-related error [2]. While the first kind of errors mainly originates from limited number of autocalibration signal (ACS) lines and restricted kernel size, the second kind of errors generates from noise in measured data and propagation error in kernel weight estimation. Some methods have been proposed to improve GRAPPA in recent years like using localized coil calibration and variable density sampling [3], multicolumn multiline interpolation [4], regularization [5, 6], reweighted least square [7], high-pass filtering [8], cross-validation [2], iterative optimization [9, 10], IIR GRAPPA [11], and so forth.

The conventional GRAPPA [1] can be considered as a *k*-space interpolation procedure along 1D phase-encoding direction, before which a fitting procedure calculates the interpolation coefficients using ACS lines. Multicolumn multiline interpolation (MCMLI) GRAPPA [4] extended the conventional GRAPPA to 2D version by fitting coefficient and interpolating missing data along phase-encoding and frequency-encoding directions, which can be viewed as improving model by increasing the kernel size. However, interpolation weights are still generated from ACS lines which are contaminated by noise in sampling from scanner, so that noise still exists, which is likely even to be exaggerated in propagation process. Furthermore, as reduction factor increases, image quality deteriorates severely by noise and residual aliasing [8]. On the other hand, some datasets were seriously noise contaminated, so they display poor SNR even at low reduction factor around 2 or 3.

Noise has been a major concern in many MRI experiments. Some researchers also studied GRAPPA noise problem from various angles, including truncated SVD and Tikhonov regularization [5] and iterative reweighted least square [7]. These methods tried to suppress noise in estimating weight process rather than from system identification perspective. Since GRAPPA reconstruction has been viewed as a linear system [1, 11], in which ACS and a part of acquired data construct the input and output of the system in fitting process, based on the observation that input and output have been contaminated by noise from scanner, estimation of GRAPPA modeling weights will be biased, whose severity depends on measured noise power. This paper studies GRAPPA as a noisy input-output system, which has been addressed in various ways, including Koopmans-Levin (KL) method [12], logarithmic least squares frequency-domain method [13], combined instrumental variables and subspace fitting method [14], and bias-eliminated least squares methods [15]. Noisy input-output system can be described by means of errors-in-variables (EIV) model [13, 16], which accounts for measurement errors in parameter estimation [17]. Since noisy signals exist in inputs and outputs of fitting procedure, GRAPPA is generalized as a noisy input-output system represented by the EIV model.

This article presents a framework based on the EIV model for identifying true weights of GRAPPA reconstruction. Under this framework, a concrete method—IV GRAPPA—is proposed, which discovers true functional relationship among sampled and missing *k*-space signals in terms of accurate fitting weights. The proposed method provides a practical approach for improving SNR with good performance. In the following sections, we provide theoretical foundation and mathematical description of the proposed method, and based on which, a set of representative experimental results and our discussions are presented.

## 2. Theory and Method

### 2.1. GRAPPA

The key component in GRAPPA is a segmented fitting and interpolation routine [4]. Central *k*-space of each coil is fully sampled at the Nyquist rate, and outer *k*-space is downsampled by a reduction factor. Central *k*-space, as the training dataset, is used for estimating weights, and then missing data points are reconstructed by a linear combination of acquired points from all *L* coils. This fitting and interpolation processes can be generalized as the following equation:

(1) Sjky+rΔky,kx∑l=1L ∑b=−NbNa ∑h=−HlHrwj,rl,b,h·Slky+bRΔky,kx+hΔkx,

where *S* is *k*-space signal, *w* denotes weight set, *R* denotes reduction factor, *j* is the target coil, *l* counts all coils, and *b* and *h* transverse neighbor acquired points. The variables *k*_*x*_ and *k*_*y*_ represent coordinates along frequency-encoding and phase-encoding directions, respectively.

The formulation of GRAPPA can be generalized as an overdetermined system of linear equations [5, 11, 18], which can be simply represented as

(2) $$\begin{array}{c} b=Ax, \end{array}$$

where *A* represents the acquired data matrix in fitting and interpolation processes which is the same as *S*_*l*_ in (1), *b* denotes acquired data matrix in fitting process and target data matrix in interpolation process which is equivalent to *S*_*j*_ in (1), and *x* represents reconstruction weights which is equivalent to *w* in (1). From the linear system view, *A* and *b* represent input and output of the system, respectively. The objective of the fitting process is to calculate the interpolation weights, and interpolation process maps the acquired data to a desired complete set of *k*-space data.

### 2.2. Noisy Input-Output System

In the fitting process, since both input *A* and output *b* are acquired from central *k*-space, which have the measurement noise, GRAPPA is belonged to errors-in-variables (EIV) problem [17]. Linear inverse problems give rise to parameter estimation problem with correlated errors-in-variables, in which both input and output variables are contaminated by noises, so fitting such data using standard least squares can lead to bias in the solution.

Usually measurement errors are usually described using latent variables approach [19]. If *A* and *b* are observed values of acquired *k*-space signal, we assume that there exist some unobserved latent variables (noise-free signal) *A*′ and *b*′, which model the true functional relationship *f* of fitting and interpolation, such that observed values *A* and *b* are contaminated noisy observation:

(3) A=A′+ηA,b=b′+ηb,f:b′=A′x′,

where *η*_*A*_ and *η*_*b*_ represent contaminated noises and *x*′ denotes true fitting weights, which cannot be observed.

In the fitting process, both *A* and *b* are known variables; according to (2) and (3), *f* : *b*′ = *A*′*x*′ can be reformulated as

(4) $$\begin{array}{c} b^{\prime }+\eta_{b}=\left(\;A^{\prime }+\eta_{A}\;\right)x, \end{array}$$

where all denotations have the same meanings as those of (2) and (3). So, there is a bias *η*_*x*_ in weights *x* generated from fitting process:

(5) $$\begin{array}{c} x=x^{\prime }+\eta_{x}. \end{array}$$

In the interpolation process, target data are unknown variables denoted by *b*_*u*_, which can be generalized as

(6) buAx=A′+ηAx′+ηx=A′x′+A′ηx+ηAx′+ηAηx,

where *η*_*A*_*η*_*x*_ represents the propagated noise errors in terms of a nonlinear form. In addition, the terms *A*′*η*_*x*_ and *η*_*A*_*x*′ also contribute biases in estimating the target signal *b*_*u*_. From above analysis, we can see noise generation routine in GRAPPA under the framework of errors-in-variables problem.

### 2.3. IV GRAPPA

In order to reduce noise level caused by EIV problems, some methods have been proposed, including total least squares (TLS) and instrumental variables [16]. TLS method that is a modeling technique with considering observational errors is not scale invariant [20], so that it may not be directly used for removing noise in *k*-space reconstruction, because signal amplitudes at low frequency and high frequency of *k*-space differ largely in scale.

The IV method is commonly used to estimate the system dynamics (the transfer function from the input *A* to the output *b*) [16], which provides a consistent estimator when explanatory variable (such as *A* in (3)) is correlated with error terms (such as *η*_*A*_ in (3)). It needs to provide an instrument weighting matrix. To achieve consistent estimation, instruments variables are uncorrelated with errors and correlated with endogenous explanatory variable (such as *A*′ in (3)). Since least squares method generally achieves biased and inconsistent estimates where measurement errors exist, IV method may achieve unbiased and consistent estimates of fitting weights in GRAPPA. According to model structure in (2) for estimating weights *x*, while least squares method is formulated as

(7) $$\begin{array}{c} {\hat{x}}_{LS}={\left(\;AA^{H}\;\right)}^{-1}Ab, \end{array}$$

the basic IV estimate of *x* is presented as

(8) $$\begin{array}{c} {\hat{x}}_{IV}={\left(\;ZA^{H}\;\right)}^{-1}Zb, \end{array}$$

where *Z* represents instrument matrix and *H* denotes conjugate transpose. The generalized method of moments (GMM) [21] can be used to generate IV estimator as

(9) x^IVAHZZHZ−1ZHA−1AHZZHZ−1ZHb=AHPZA−1AHPZb,

where *P*_*Z*_ represents the projection matrix *Z*(*Z*^*H*^*Z*)^−1^*Z*^*H*^. This IV estimator is also called the generalized instrumental variables estimator (GIVE), or the two-stage least squares (2SLS) estimator [22].

Based on IV estimator, we can apply it on GRAPPA reconstruction for estimating “true” weights. However, as mentioned above, model error also exists in GRAPPA reconstruction, which is not conformed to EIV model. For this reason, in actual experiments, model error is also exaggerated by IV method so that aliasing artifacts are more obvious than that generated by least squares method. We only consider removing noise-related error here using IV estimator in (9).

### 2.4. Selection of Instrument

There are two main requirements of choosing instruments [22]:

- The instruments should be correlated with the endogenous explanatory variables, conditional on other explanatory variables.
- The instruments cannot be correlated with the error terms (noise).

For *k*-space, because noise-related errors are generally decided by signals at high frequency region (outer region) and generation of aliasing artifacts is usually dependent on signals at low frequency region (central region), in order to avoid aliasing artifacts' deterioration by IV method mentioned above, we choose signals between high frequency and low frequency regions for constructing instruments to achieve a compromise between removing noise and suppressing aliasing artifacts.

One common method of selecting instruments is to take the delayed outputs of the system [16]. In fitting process as shown in (2), the outputs of linear fitting process are still ACS data and acquired data sampled from *k*-space. Therefore, we directly use a part of these ACS data and acquired data to construct the instruments *Z* for solving weights, which can be considered as the delayed outputs of the system. Specifically, we usually choose 2000–4000 data points on ACS and acquired lines between low and high frequency regions as instruments in experiments for 256 × 256 size *k*-space. Furthermore, we also defined a central window on *k*-space, in which missing data points are still interpolated by weights generated from the conventional least squares technique. On the other hand, missing points outside of the central window are generated by IV method. After interpolating missing points, some of which are then used to calculate goodness-of-fit coefficients to combine sliding blocks [1], ACS lines and acquired lines are finally used to replace corresponding locations to generate the complete reconstructed *k*-space.

## 3. Experiments and Results

### 3.1. Experiment Settings

The performance of the proposed method is validated by four datasets. A phantom was sampled by Gradient Echo pulse sequence with parameters (TE/TR = 10/100 ms, 31.25 kHz bandwidth, matrix size = 256 × 256, FOV = 250 mm^2^) on a 3T commercial scanner (GE Healthcare, Waukesha, WI) with an 8-channel head coil. The second is a four-channel head coil (axial plane, 4 coils, 256 × 256 matrix) scanned on a 3T commercial scanner (GE Healthcare, Waukesha, WI). The third is a sagittal dataset that was on a 1.5T SIEMENS Avanto system with a 4-channel head coil using a 2D T1-weighted spin echo protocol (TE/TR = Min Full/500 ms, 24 cm FOV, 256 × 256 matrix). The fourth is a set of in vivo brain data, which were acquired on a 3T commercial scanner (GE Healthcare, Waukesha, WI) with an 8-channel head coil (In vivo, Gainesville, FL).

The sum-of-square (SoS) reconstructions from the fully sampled data of all channels are shown as the reference (denoted as “Ref.” in images) for comparison. GRAPPA reconstructions with the same sampling pattern and with the same net reduction factor are also presented for comparison. For each dataset, all images are shown in the same scale. For kernel size, the number of blocks usually takes 4, and the number of columns ranges from 1 to 10 [1, 4, 8, 9, 11]. We also choose 4 blocks, and a larger number of columns: 11 for reducing the influence of artifacts.

### 3.2. Results and Discussion

In the case of phantom data to which white Gaussian noise was added, the reduction factor takes 4 for GRAPPA reconstruction. For the equivalent comparison, the proposed method adopts the same sampling pattern as above GRAPPA reconstruction with reduction factor 4, 4000 acquired and ACS data points, and a 32 × 32 central window are used. Again, GRAPPA reconstruction with the same net reduction factor is also considered for comparison, in which a lower reduction factor of 3 and less number of ACS lines are used.

For the phantom dataset, we can see that, at reduction factor 4, noises are generated by GRAPPA reconstruction, which seriously deteriorate image quality. Furthermore, another sampling pattern of reduction factor 3 and 32 ACS lines, which has the same net reduction factor as the previous sampling pattern, also has noise contamination problem. By comparison, the proposed IV method better removes the noise in reconstruction process, which reduces more noise than both GRAPPA reconstructions.

Figure 2 shows experimental results for the four-channel brain (axial), the four-channel brain (sagittal), and the eight-channel brain (axial) datasets, respectively. For the four-channel brain (axial) dataset, the reduction factor also takes 4 for GRAPPA reconstruction, and the proposed method adopts the same sampling pattern with reduction factor 4, 2000 acquired and ACS data points for instruments, and a 64 × 64 central window. In addition, GRAPPA reconstruction with the same net reduction factor is also considered for comparison, in which a lower reduction factor of 3 and less number of ACS lines are used. For the four-channel brain (sagittal) dataset, the sampling pattern with the reduction factor 3 and 64 ACS lines is used for GRAPPA and the proposed method that adopts 4000 acquired and ACS points for instruments and a 64 × 64 central window. Similar to four-channel brain (axial) dataset, the eight-channel brain (axial) dataset uses almost the same sampling pattern to that of the four-channel brain (axial) dataset. The proposed method uses 4000 acquired and ACS data points for instruments and a 48 × 48 central window for hybrid least squares and IV estimation.

Visual evaluation on three in vivo dataset results indicates that compared to GRAPPA reconstructions with the same sampling pattern and with the same net reduction factor, the proposed method improves the image quality in terms of SNR and preserves more details. Furthermore, the proposed method also suppresses the aliasing artifacts well. The processing time of the proposed method is about 1.5–3 times of GRAPPA reconstructions.

**Figure 1:**
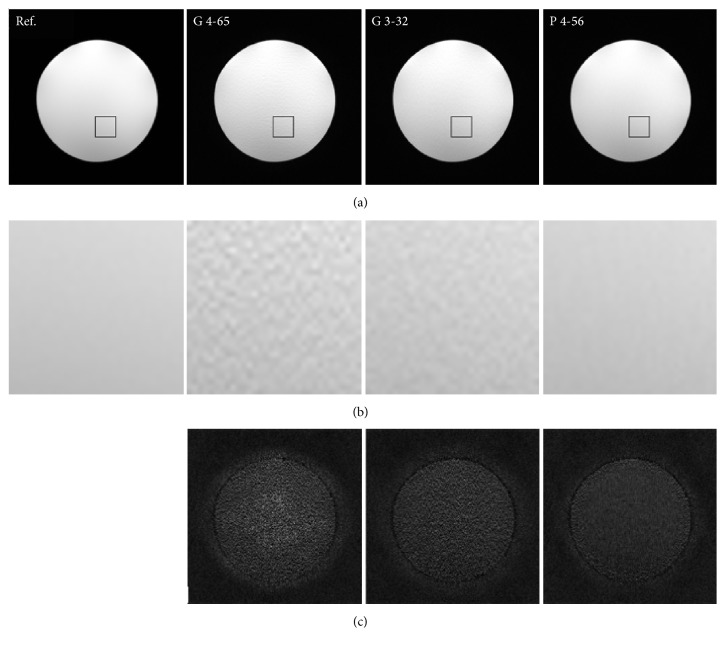
The reconstructions (a), zoomed square regions (b), and difference maps (c) from scanned phantom data, in which each column represents one kind of reconstruction or reference. “G” represents GRAPPA, “P” denotes proposed method, the number at left of “-” represents reduction factor, and the number at right of “-” is the number of ACS lines.

**Figure 2:**
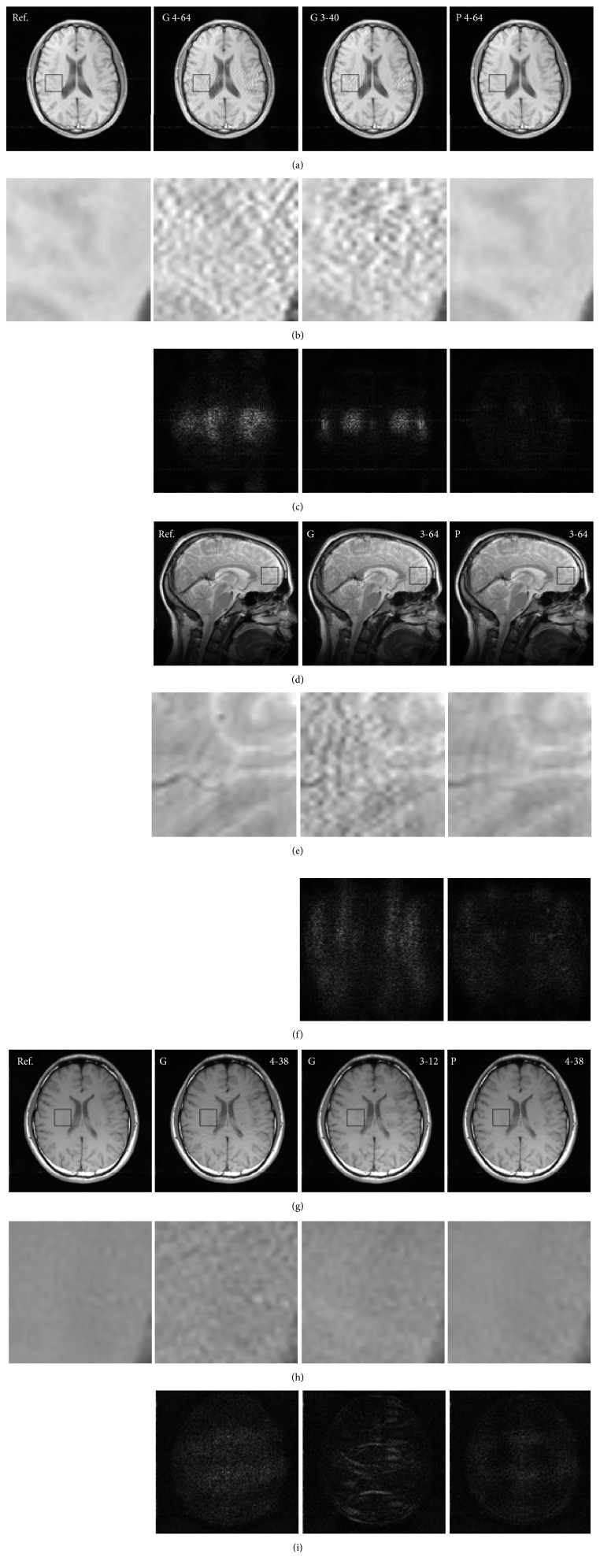
The reconstructions (rows (a), (d), (g)), the corresponding zoomed square regions (rows (b), (e), (h)), and difference maps (rows (c), (f), (i)) from scanned in vivo data, in which “Ref.” is reference image, “G” represents GRAPPA, and “P” denotes proposed method, the number at left of “-” represents reduction factor, and the number at right of “-” is the number of ACS lines. Rows (a–c) are the four-channel brain (axial) results; rows (d–f) are the four-channel brain (axial) results; rows (g–i) are the eight-channel brain (axial).

The normalized mean square error (NMSE) provides a quantitative evaluation on experimental results as shown in Table 1. The parameters of reconstructions in Table 1 are the same as previous ones for Figures 1 and 2. In terms of NMSE, the proposed method is superior to both GRAPPA reconstructions for each testing dataset.

## 4. Conclusion

In this paper a general framework for removing noise of Cartesian GRAPPA reconstruction is presented. The noise generation procedure can be derived from the noisy input-output system perspective for fitting and interpolation components of GRAPPA. The IV method that has been successfully used to solve the EIV problems is applied on GRAPPA reconstruction to estimate “true” weights. Under the proposed framework, it may be possible for other methods (such as [12–15]) that solve the EIV problems to remove noise introduced in GRAPPA reconstruction.

## Figures and Tables

**Table 1:** Comparison of NMSEs.

	GRAPPA with same sampling pattern	GRAPPA with same reduction factor	Proposed
8-coil phantom	0.07	0.0421	0.0326
4-coil axial	0.4704	0.2506	0.0576
4-coil sagittal	0.6121	None	0.246
8-coil axial	0.1951	0.1176	0.1079
